# Study of nerve cell regeneration on nanofibers containing cerium oxide nanoparticles in a spinal cord injury model in rats

**DOI:** 10.1007/s10856-023-06711-9

**Published:** 2023-02-21

**Authors:** Behnaz Rahimi, Zahra Behroozi, Ali Motamednezhad, Maral Jafarpour, Michael R. Hamblin, Ali Moshiri, Atousa Janzadeh, Fatemeh Ramezani

**Affiliations:** 1 Department of basic sciences, Saveh University of Medical Sciences, Saveh, Iran; 2 Physiology Research Center, Institute of Neuropharmacology, Kerman University of Medical Science, Kerman, Iran; 3 Radiation Biology Research Center, Iran University of Medical Sciences, Tehran, Iran; 4 International Campus, Iran University of Medical Sciences, Tehran, Iran; 5 Laser Research Centre, Faculty of Health Science, University of Johannesburg, Doornfontein, 2028 South Africa; 6 Dr. Moshiri Veterinary Clinic, Tehran, Iran; 7 Physiology Research Center, Iran University of Medical Sciences, Tehran, Iran

## Abstract

Since the CNS is unable to repair itself via neuronal regeneration in adult mammals, alternative therapies need to be found. The use of cerium oxide nanoparticles to repair nerve damage could be a promising approach for spinal cord reconstruction. In this study, we constructed a scaffold containing cerium oxide nanoparticles (Scaffold-CeO_2_) and investigated the rate of nerve cell regeneration in a rat model of spinal cord injury. The scaffold of gelatin and polycaprolactone was synthesized, and a gelatin solution containing cerium oxide nanoparticles was attached to the scaffold. For the animal study, 40 male Wistar rats were randomly divided into 4 groups (*n* = 10): (a) Control; (b) Spinal cord injury (SCI); (c) Scaffold (SCI + scaffold without CeO_2_ nanoparticles); (d) Scaffold-CeO_2_ (SCI + scaffold containing CeO_2_ nanoparticles). After creation of a hemisection SCI, scaffolds were placed at the site of injury in groups c and d, and after 7 weeks the rats were subjected to behavioral tests and then sacrificed for preparation of the spinal cord tissue to measure the expression of G-CSF, Tau and Mag proteins by Western blotting and Iba-1 protein by immunohistochemistry. The result of behavioral tests confirmed motor improvement and pain reduction in the Scaffold-CeO_2_ group compared to the SCI group. Decreased expression of Iba-1 and higher expression of Tau and Mag in the Scaffold-CeO_2_ group compared to the SCI group could be the result of nerve regeneration caused by the scaffold containing CeONPs as well as relief of pain symptoms.

**Graphical Abstract:**

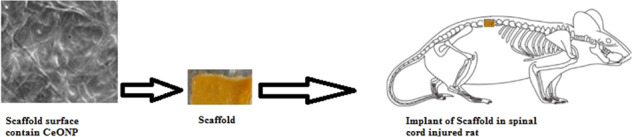

## Introduction

Damage to the central nervous system (CNS), including the brain and spinal cord due to physical injury is one of the leading causes of death and chronic disability in humans. Spinal cord injury (SCI) is typically caused by axonal damage, resulting in nerve cell and glial cell death [1–3]. Secondary outcomes of SCI including uncontrolled inflammation, nerve irritability, edema, ischemia, free radical production, cell death (apoptosis), severe glutamate overstimulation, and chronic demyelination with glial scar formation prevent any axonal regeneration and cause subsequent neuropathic pain [1, 2]. Spinal cord injury affects the motor, sensory, and even the autonomic nervous systems, causing motor problems and reduced activity [4, 5]. These processes occur within minutes to weeks and can last years after the injury. During this period, under the influence of secondary processes, the primary injury spreads to the surrounding healthy area on the cranio caudal axis, causing partial or complete loss of physiological function at the site of injury [6].

Complementary therapeutic approaches, including cell therapy [7, 8], glial scar digestion, neurotrophic factor delivery, laser therapy [9–11], and electrical stimulation of surrounding tissue as well as clinical rehabilitation, are being developed to achieve nerve fiber regeneration and functional restoration in SCI. But the extent of neural tissue destruction in chronic SCI in humans, with entire segments of the spinal cord replaced by fluid-filled cysts, remains a critical concern. In these regions, the mechanical substrates that provide physical support for axonal regeneration and the three-dimensional positional information and architectural organization required for effective nerve regrowth are permanently lost. Hence, a pressing issue in chronic SCI is to ensure an adequate level of anatomical, tissue, and cellular regeneration at the lesion site. Therefore, scar tissue and hollow cysts must be replaced with new material that allows for both axonal regrowth and bridging of the lesion. In this regard, using biodegradable implants that fill the cavities, and cause the nerve cell regeneration is recommended [12–14].

One of the most important features of successful implant integration in damaged spinal cord tissue, is its optimal mechanical strength. If the biomaterial is too rigid, it can compress the regenerating axons and create additional secondary cavities between the implant and the surrounding spinal tissue [15]. The biodegradable biomaterials used to regenerate nerve tissue are usually lost after weeks or months, depending on the growth of new axons [16–19].

Different types of scaffolds, including electrospun nanofibers, can act as a substrate for nerve cell differentiation and growth, and allow new cell-cell and cell-matrix interactions [20–22]. Nanofibers by creating a platform for drug release, prevent the cascade of secondary damage (neuroprotection), while nanofibrous structures help reestablish neural connectivity by promoting axon sprouting (neural regeneration) in order to achieve rapid functional recovery of the spinal cord [23].

Electrospun nanofibers have been widely used for skin [24, 25], bone [26] and nerve [27] tissue engineering applications due to their mimicry of extracellular matrix (ECM), biodegradability and biocompatibility. Natural and synthetic biomaterials containing PCL-gelatin have been extensively studied for tissue engineering applications. The combination of the biological properties of natural polymers and the physicochemical properties of synthetic polymers helps to overcome each other’s deficiencies. Poly ε-caprolactone (PCL) is a biodegradable polyester. Gelatin (a natural polymer derived from collagen) can be combined with PCL to facilitate cell adhesion [27, 28]. Nanofibers can also be functionalized with various agents such as drugs, growth factors or nanoparticles [24, 25].

The effectiveness of three-dimensional aligned nanofibers based on poly(ε-caprolactone) was evaluated in a hemi-incision model at the 5^th^ cervical level in rat spinal cord. In this study, aligned axon regeneration was observed as early as one week after injury, and no excessive inflammatory response and scar tissue formation was triggered [29]. In another study, a poly(ε-caprolactone)/ Polysialic acid hybrid nanofibers scaffold encapsulating glucocorticoid methylprednisolone (MP) was used to treat a transection SCI model in rats. This scaffold decreases tumor necrosis factor-α (TNF-α) and interleukin-6 (IL-6) release by inhibiting ionized calcium-binding adapter molecule 1 (Iba1) positive microglia/macrophage activation and reduces apoptosis-associated Caspase-3 protein expression. In addition, the scaffold inhibits axonal demyelination and glial fibrillary acidic protein (GFAP) expression, increases neurofilament 200 (NF-200) expression and was shown to improve functional recovery [30].

The effects of electrospun poly(ε-caprolactone)/type I collagen nanofiber conduits on the repair of peripheral nerve damage in rats treated with these electrospun nanofibers showed, no serious inflammatory reactions were observed in the hind limbs and the morphology of myelin sheaths in the injured sciatic nerve was close to normal and rats that underwent repair with electrospun nanofiber conduits tended to have greater sciatic nerve function recovery [31].

Recently, study of metal nanoparticles has become the focus of intense research due to their unusual properties compared to bulk metals, especially since they are used either to inhibit the growth of microorganisms [32–35], cancer cells [36], or to stimulate the growth of plant [37, 38] and animal cells [39, 40] and also in this way affect the production of many intermediate molecules [41]. Researchers have shown that the metal nanoparticles, that can act as anchors to the substrate, improve nerve-to-substrate interactions, leading to controlled nerve cell growth [42, 43]. Cerium oxide nanoparticles (CeONPs) promote neuronal differentiation and increase neuronal survival [44]. Cerium oxide nanoparticles have excellent catalytic activity due to the redox conversion between Ce^3+^ and Ce^4+^ states [45]. Cerium oxide nanoparticles appear to mimic the activity of superoxide dismutase, catalase, peroxidase, and various oxidase enzymes, as well as possessing the ability to adsorb hydroxyl radicals and nitric oxide [46]. Because of these properties, CeONPs are used as antibacterial agent [33], anticancer agent [47] and also as a neuroprotective agent that can help reducing neuron damages after injury [44, 48]. Attilio Marino in 2017 showed the positive effect of cerium oxide-gelatin nanofibers on the differentiation of SH-SY5Y bone marrow cells into neuron-like cells. This group found that the antioxidant activity of CeO_2-_nanofibers was effective in cellular differentiation [49]. Ciofani in 2013 showed that CeONPs could differentiate neuron-like cells from PC12 cells and confirmed the potent antioxidant activity of CeONPs [50].

Dong et al. in 2020, in an in-vitro spinal cord model system demonstrated the biofabricated nano-cerium oxide loaded poly (e-caprolactone) (PCL)/resveratrol (RVL) treatment significantly preserved hydrogen peroxide and also good catalytic performance [51]. In study by Wang et al in 2021, selenium NPs encapsulated CeO_2_ nanostructures administrations for SCI therapies have greatly suppressed oxidative stress and induced anti-inflammatory action, which leads to prospective therapeutic benefits of spinal cord regeneration [52].

Kim et al. also showed that CeONPs had an antioxidant effect in spinal cord injury and subsequently improved the functional recovery in rats after mild traumatic brain injury [53]. In last work our team also demonstrated the healing effect of soluble CeONPs on neuronal regeneration after SCI [54] but at the present study, the release of nanoparticles was continuously from the fabricated scaffold, and the novelty of this study lies in this issue. In the present study, we investigated the effect of a gelatinous poly(ε-caprolactone) scaffold containing CeONPs (Scaffold-CeO_2)_ implanted at the site of injury on nerve cell growth and pain relief in a SCI animal model.

## Material and methods

### Preparation of scaffold

Chloroform (8 mL) and 0.4 g of PCL were combined and then mixed with 0.16 g of gelatin and 2 mL of 80% acetic acid. The resulting solution was mixed for 3 h to form a jelly-like structure. The mixture was then refrigerated for 48 h to obtain a flexible integrated scaffold. The dissolved CeONPs (1000 µg/mL, purchased from Sigma Aldrich, 20 wt. % in H_2_O, pH~ 4, ID: 796077) in gelatin-acetic acid was electrosprayed onto the scaffold with 60% power for 1 h according to the following protocol. The gelatin solution containing the nanoparticles was rotated at 30 °C using a voltage of 20 kV, a flow of 10 μL/min and a nozzle distance of 10 cm to produce fibers on the scaffold. A fixed axis was used to concentrate the fibers at one point. The Scaffold-CeO_2_ was characterized via Energy Dispersive X-ray (EDX) and Scanning electron microscopy (SEM). After coating the samples with gold, the final Scaffold-CeO_2_ structure was imaged using a scanning electron microscope (SEM, DSM-960A Zeiss, Carl Zeiss, Germany). Energy Dispersive X-ray (EDX system Kevex) spectroscopy was performed to identify the elements in the nanofiber.

### In vitro release of CNPs

Investigation of the release of CNPs from the Scaffold-CeO_2_ was similar to our last published article [33]. In summary, the Scaffold-CeO_2_ was immersed in PBS at 37 °C for 9 days. The optical density of the samples was measured at 300–350 nm using a UV–Vis spectrophotometer (Thermo Fisher Scientific, Waltham, Massachusetts, USA) on days 1, 3, 5, 7, and 9. Experiments were performed in triplicate.

### Animal study

Scaffold-CeO_2_ were implanted in rats suffering from spinal cord lesions. In this study, male Wistar rats weighing about 200–250 g were used and randomly divided into 4 groups (*n* = 10). The animal experiments were approved by IRAN University of Medical Sciences ethics approval center with COD number IR.IUMS.REC.1398.318

- Control; without any surgery or treatment
- SCI; Spinal cord injury induced without any treatment
- Scaffold; SCI group with an implant of scaffold without CeONPs
- Scaffold-CeO_2_; SCI group with an implant of scaffold containing CeONPs

To induce the SCI hemisection model, the animal was anesthetized and after locating the desired site at T12 to T13 vertebral level which is equal L2-L3 of the spine, the skin and muscle were separated and the vertebrae were broken with rongeur in this location. After observing the spinal cord, the upper layer of the spinal cord was cut with microdissection scissors for creating the hemisection model. The gap produced had a width of 2 mm and was removed with a 22-gauge needle [55]. The removed spinal cord was replaced with Scaffold or Scaffold-CeO_2_. At 7 weeks, motor function and behavioral experiments were performed on the animals. According to other studies, 7 weeks is sufficient time to investigate the regeneration of axon and glial cells after SCI [56, 57]. Perfused and fresh tissues were prepared to measure the expression of G-CSF, Tau, Mag, and Iba-1.

### Behavioral tests

#### Basso, Beattie, Bresnahan (BBB) scores

The animals were placed in a circular space one meter in diameter and their behavior was studied for 4 min. This test was performed by two blinded investigators weekly. In summary, the motor behavior of the animals included the following components: hind limb movement, animal weight bearing, limb coordination, and walking. According to the instructions, the animals were given grades from 0 to 21 [58].

#### Thermal hyperalgesia (Radiant heat)

The sole of the rat foot was used to measure the heat pain threshold. For this purpose, the animals were placed in a plexiglas container and infrared radiation was delivered through the bottom of the container onto the animal paw. The animals were given 15 min to adjust to the environment, and then infrared was irradiated onto the bottom of the animal paw. Removing the paw automatically stopped the heat generated by the infrared source. To prevent burn damage, a cut-off of 25 sec was used by the observer. This test was performed 3 times on each paw at intervals of at least 1-minute, and the average of the obtained numbers was calulated as a response.

#### Von frey filament allodynia measurement test

Von Frey filaments were used to measure mechanical allodynia [59]. For this purpose, pressure was applied to the sole of the animal foot by filaments of different thicknesses and the animal foot withdrawal response was measured. To test the animal, it was placed in a net cage about 30 cm above the ground and von Frey filaments with numbers 4.08, 4.31, 4.56, 4.74, 4.93, and 5.18 were applied. The results were evaluated using the up and down method. In this method, first the filament number 4.31 was used, and progressively thicker filaments were used in case of no response or thinner filaments were used in case of a positive response. Finally, the data were analyzed by Dixon software.

#### Cold Allodynia Test (Acetone test)

The animals were placed in special cages with a net floor that was 30 cm above the bench surface. Fifteen minutes after placing the animal in the cage, a drop of acetone was injected into the sole of the animal foot, and the animal reaction, including the foot reflex, licking, or foot shaking was examined. This procedure was performed 5 times for each leg at intervals of at least one minute, and finally the animal reaction was calculated as a percentage [59].

#### Tissue evaluation

Rats were anaesthetized with (ketamine 100 mg/kg, and xylazine 10 mg/kg, IP). Transcardial perfusion was used to fix the spinal cord. First, normal saline was injected into the heart to remove blood from the spinal cord, and then 4% paraformaldehyde in 0.1 M phosphate buffer (pH = 7.2–7.4) was perfused to fix the spinal cord. The fixed spinal cord was dissected and post-fixed in 4% paraformaldehyde for 48 h, and then blocked with paraffin. Section 5 μm in thickness were cut for tissue staining [60].

#### Histological Study

To determine the cavity size in the spinal cord after injury by Hematoxylin and Eosin staining (H&E), the longitudinal sections of the spinal cord on the site of injury were stained by H&E staining (*n* = 3 per group). For this reason, the sectioned were deparaffinized and rehydrated by a series of graded alcohols and stained with H&E stain according to the manufacturer’s guidance [60]. Three sections from the T12-T13 level of the spinal cord, were chosen in each animal. Pictures were captured from the sections by Olympus microscope with objective magnification of 4 and the cavity size was assessed via Image J software. According to prior studies [61], the cavity size was calculated by the following formula:

$${\rm{The}}\,{\rm{percentage}}\,{\rm{of}}\,{\rm{cavity}}\,{\rm{size}} = \left( {{\rm{Cavity}}\,{\rm{size}}\,\left( {\mu{\rm{{m}}}} \right)} \right)/\left( {{\rm{Total}}\,{\rm{area}}\,{\rm{of}}\,{\rm{the}}\,{\rm{section}}\,\left( {\mu{\rm{m}}} \right)} \right) \times 100$$

Nissl staining was used to distinguish healthy neurons from dead neurons [62]. Mason Trichrome Staining (MTS) was used for selective differentiation visualization of collagen fibers, according to the usual protocol [63]. After the Nissl staining, images were captured (Olympus, magnification × 4 and x 40) and the number of the dead cell around the injury site was calculated via ImageJ software. All experimental groups took images from a specific area in longitudinal sections of the spinal cord. The length of the spinal cord was about one centimeter, and the photos were taken from the spinal cord’s dorsal surface in the middle area, an area at the L2-L3 spinal level. Five fields in each section were randomly selected, and the mean number of dead cells was calculated. Data analysis was carried out using Graph Pad Prism version 7.03. One-way analysis of variance (one-way ANOVA) as Bonferroni post hoc test were used in order to compare between different groups. All results were showed as mean ± standard deviation and *p* < 0.05 was considered significant.

#### Immunohistochemistry

For immunohistochemistry, the slides were incubated at 60 °C for 3 h. The slides were immersed in two changes of 100% xylene then graded ethanol concentrations. Blocking was then performed with 5% hydrogen peroxide solution in methanol and washed twice in TBS. 100 μL of the diluted Iba-1 primary antibody (1/100, orb10863) was added to the slides and incubated in a humidified container at room temperature for 12 h. After washing, 100 μL of diluted horseradish peroxidase-conjugated secondary antibody was added and incubated in a humid chamber at room temperature for 30 min. The slides were washed and then 100 μL of Diaminobenzidine (DAB) solution and 0.05 mL H_2_O_2_ was added to the sections on the slides to produce the color, and the slides were observed under an optical microscope.

### Western Blotting

Seven weeks after injury, the animals (*n* = 3 in each group) were anesthetized with ketamine and xylazine. Radio immunoprecipitation assay (RIPA) buffer was added to the tissues. Tissues were centrifuged (13,000 g, 30 min, 4 °C) and supernatants were isolated for Western blotting. The lysates containing 50 μg of protein were electrophoresed on a sodium dodecyl sulfate acrylamide gel and the proteins were transferred to polyvinylidene-fluoride membranes (PVDF). After blocking, the membranes were exposed to primary antibodies including anti-GCSF antibody (orb308858, MW: 90 kDa.), total Tau antibody (1/1000, orb158145, MW: 52/79 kDa), total MAG (1/1000, orb536682, MW: 63 kDa), and β-actin antibody (1: 500, sc-47778, MW: 45KD). The membranes were then washed with TBST and incubated with horseradish peroxidase conjugated goat anti-IgG (1/1000, sc-516102). Protein bands were detected by enhanced chemiluminescence (ECL). The results were quantified by Image J software [54].

### Statistical analysis

The BBB score and neuropathic pain data were analyzed using 2-way repeated-measure ANOVA (Bonferroni post hoc test). Also, if the data were parametric, one-way ANOVA parametric tests were used for statistical analysis. Data obtained from various experiments were analyzed by SPSS 21 software. The data were expressed as mean ± SEM. *P* < 0.05 was considered significant and graphs were drawn by Excel software.

## Results

### Characterization of scaffold-CeO_2_

**Fig. 1:**
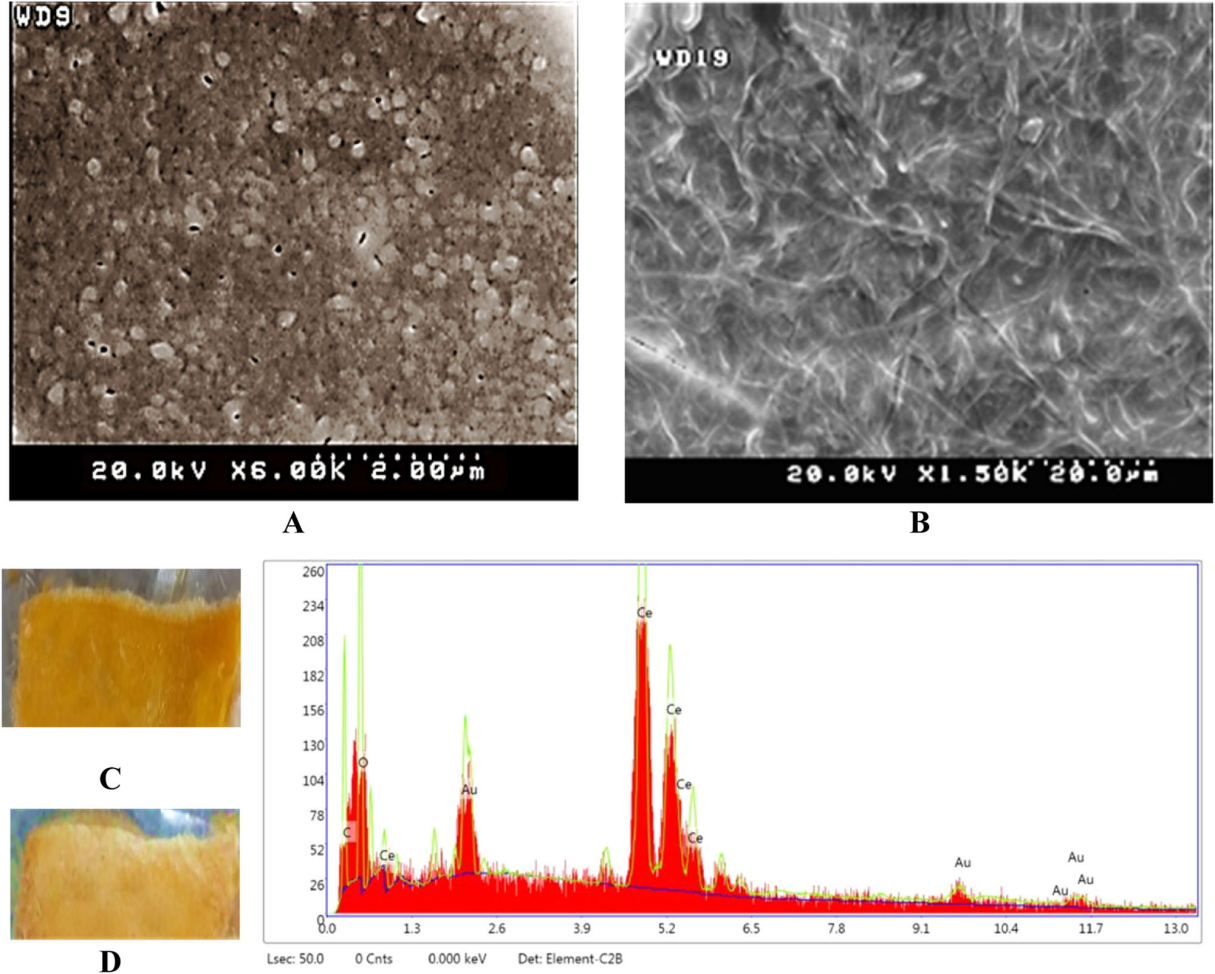
Characterization of Scaffold-CeO_2_. **A** Electron microscopy image of CeONPs. **B** Scaffold-CeO_2_ surface containing CeONPs. **C** Appearance of scaffold before electrospinning of CeONPs. **D** Scaffold after electrospinning of CeONPs. **E** Graph of EDX of CeONPs showing the peaks of cerium nanoparticles

CeONPs were characterized using electron microscopy images (Fig. 1). The images confirmed that the nanoparticles were spherical and in a size range of 5–10 nm (Fig. 1A). The images of the Scaffold-CeO_2_ are shown in Fig. 1B. Figure 1C shows the appearance of the scaffold before the electrospinning of CeONPs, and Fig. 1D shows the Scaffold-CeO_2_. Only difference between the scaffolds after the nanoparticle is sprayed on, is the presence of nanofibers with very thin diameters, which makes the surface of the substrate brighter than before. Peaks in Energy-dispersive X-ray spectroscopy (EDX) confirmed the presence of CeONPs (Fig. 1E). A release ranging between 25 and 35% was measured on day 1, with a concomitant increase to 80–90% measured on day 9 (published data in our last study) [33].

### Behavioral test

**Fig. 2:**
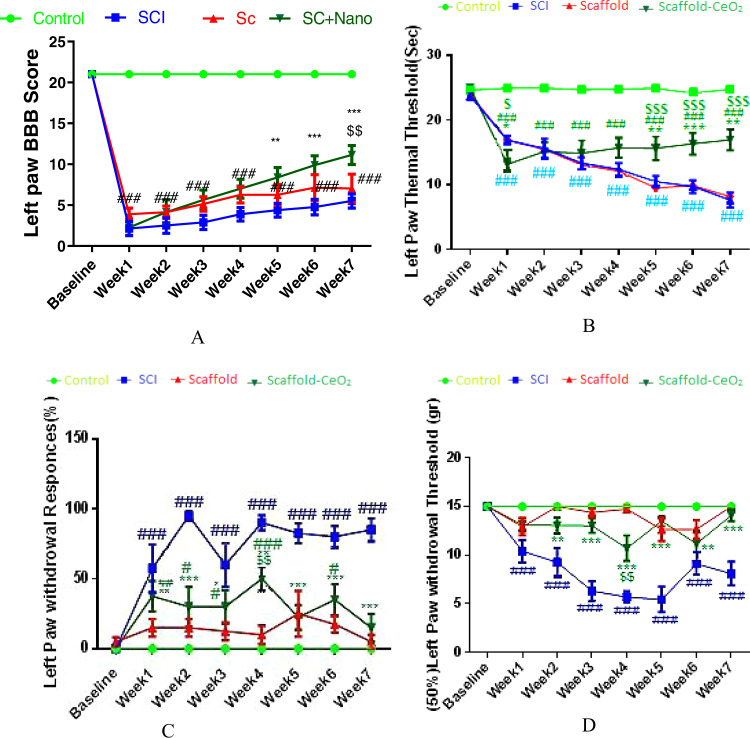
The effect of Scaffold-CeO_2_ and Scaffold implants immediately after SCI induction on left paw functions. **A** Motor function (BBB), (**B**) Thermal hyperalgesia, (**C**) Cold allodynia, (**D**) Mechanical allodynia. Data is presented as mean ± SEM (*n* = 8). In each group *p < 0.05, **p < 0.01, ***p < 0.001, ****p < 0.0001 vs. SCI group. ^#^*p* < 0.05, ^##^*p* < 0.01, ^###^*p* < 0.001, ^####^*p* < 0.0001 vs. control group. ^$$^*p* < 0.01 comparing Scaffold and Scaffold-CeO_2_. Blue; SCI Spinal Cord Injury, Red; Scaffold, Dark Green; Scaffold-CeO_2_

In the present study, seven weeks after Scaffold-CeO_2_ implantation, one rat died and was replaced in the study. Data from 40 animals were finally analyzed. The results showed that the induction of SCI hemisection during the 7-week experiment caused in a decrease in the BBB score. Significant reduction in motor function in the left paw of all SCI animals started from the first week (2.2 ± 0.53) and continued until the end of the study (6.3 ± 0.26) compared to the control group (*p* < 0.0001). The BBB score after 7 weeks in scaffold-receiving rats (6.8 ± 1.8) was similar to the untreated SCI animals (6.3 ± 0.26) (Fig. 2A). The movement of animals in Scaffold-CeO_2_ group (7.1 ± 0.9) was similar to the SCI (3.8 ± 0.) and Scaffold groups (6.0 ± 1.1) up until the fourth week (*p* < 0.001). But from the fifth week onwards, a significant improvement in movement and BBB score was observed compared between the SCI (4.2 ± 1.0) and Scaffold-CeO_2_ groups (8.4 ± 1.2). In the sixth and seventh weeks, movement in the Scaffold-CeO_2_ group was significantly improved compared to the SCI group (*p* < 0.01, *p* < 0.001). In the seventh week, in addition to the difference between the Scaffold-CeO_2_ group (11.2 ± 1.1) compared to the SCI group (5.5 ± 1.1) (*p* < 0.001), there was also a difference in the BBB score between the Scaffold-CeO_2_ group compared to the Scaffold group (6.8 ± 1.8) (*p* < 0.01).

Although SCI induction was performed on the left side of the animal spinal cord, the result of functional recovery testing on the right side was also affected and reduced movement was observed (Figure 1A of Supplementary). Significant reduction in motor function in the right paw of all SCI animals continued until the end of the study compared to the control group (*p* < 0.0001). In the Scaffold and Scaffold-CeO_2_ groups, motor improvement was seen from the first week to the end of the study. In the seventh week, only the SCI group was different from the control group) *p* < 0.001) and there was no difference in the movement of the other groups (Scaffold and Scaffold-CeO_2_) compared to the control group.

The results of thermal hyperalgesia test showed that SCI induction reduced the pain threshold of thermal hyperalgesia in comparison with the control group (*p* < 0.001). Significant reduction of the thermal pain threshold in SCI animals started from the first week (16.7 ± 0.6, *p* < 0.001) and continued until the end of the study compared to the control group (In the 7th week, 6.6 ± 1.2, *p* < 0.001). In the group treated with Scaffold, pain was observed from the first week compared to the control group (16.7 ± 0.3, *p* < 0.001), which was similar to the SCI group with an increasing slope until the end of the study. In the first week after surgery, animals receiving Scaffold-CeO_2_ experienced more pain (13.1 ± 1) than SCI (16.7 ± 0.6, *p* < 0.05). However, from the fourth week onward, an improvement in the pain threshold was observed in the Scaffold-CeO_2_ group (15.3 ± 1.4). In the fifth week (15.2 ± 1.4) until the end of the study (16.1 ± 1.8), a significant difference was observed between both groups receiving Scaffold and Scaffold-CeO_2_ and the SCI group. However, the pain threshold did not reach the level in control rats at the seventh week (*p* = 0.001). (Figure 2B).

Removal of the left side of the spinal cord caused hyperalgesic pain in the right side of the spinal cord, which was sinusoidal in all groups (Figure 1B of Supplementary). Pain was observed in the SCI group during the study compared to the control group (*p* < 0.001). Also, in the other treatment groups, more pain was observed during the study compared to the control group (*p* < 0.001). In the Scaffold-CeO_2_ group, pain decreased in the seventh week (18.6 ± 1.3) compared to SCI group (12.8 ± 1.1) and the Scaffold-treated group (13.1 ± 1.8) (*p* < 0.01).

The results of cold allodynia (acetone test) (Fig. 2C) of the left paw showed that in animals with SCI, cold allodynia increased compared to the control group (*p* < 0.0001). However, both Scaffold (4.6 ± 3.3) and Scaffold-CeO_2_ (17.0 ± 8.0) treatment reduced the cold threshold in the seventh week close to the control group, and the difference between them was not significant.

In the first week after induction of SCI, pain from cold allodynia was observed in right paw of the SCI group (84.4 ± 96.0) and reduced to the end of the study (28.7 ± 9.3) (*p* < 0.001) (Figure 1C of Supplementary). At the end of the seventh week, no significant cold allodynia pain was observed in the Scaffold group (5.6 ± 5.8) compared to control in right paw. The course of regaining pain tolerance in the Scaffold-CeO_2_ transplant animals was similar to the Scaffold group, except that the pain relief began from the fifth week.

The mechanical allodynia test showed that SCI reduced the left paw withdrawal threshold (*p* < 0.0001) (Fig. 2D). Von Frey testing in the fourth week in SCI animals (5.6 ± 0.5) was significantly reduced compared to the control group (*p* < 0.001) and continued until the seventh week (*p* < 0.0001). In the Scaffold group (14.7 ± 0.2), a significant difference was observed in the seventh week compared to the SCI group (8.03 ± 1.4) (*p* < 0.001).

Induction of SCI in the left side of the spinal cord caused mechanical allodynia in the right paw of animals compared to the control group (*p* < 0.001) (Figure 1D of Supplementary). This pain was evident up to the end of the study. However, in weeks 5 and 7, the pain intensity decreased slightly and was different from the control group (*p* < 0.01). The use of Scaffold (12.5 ± 1.1) alone and Scaffold-CeO_2_ (14.6 ± 0.3) could reduce mechanical hyperalgesia in seventh week.

### Histological results

**Fig. 3:**
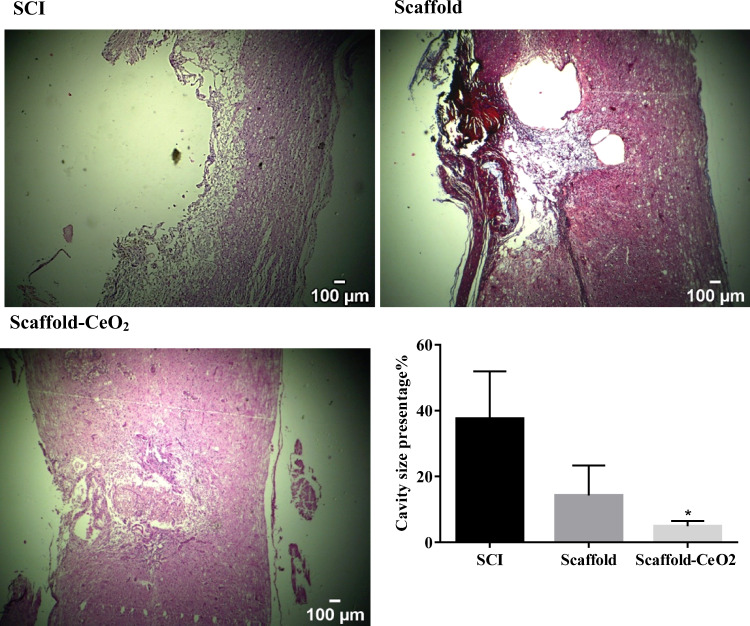
Hematoxylin and Eosin (H&E) staining for assessment of cavity size in the longitudinal sections of the spinal cord. The largest cavity was observed in the SCI group. Data are expressed as mean ± SD (*n* = 3 in each group). Original magnification ×4. *p < 0.05, versus SCI group

A large cavity in the spinal cord was identified in SCI animals group after injury. The mean cavity size was 37.55 ± 8.31 percentage in SCI and, respectively (*p* = 0.0170). The transplanted animals showed a smaller cavity in the spinal cord compared to the SCI group (*p* < 0.02). The mean cavity size in the transplanted group was 4.94 ± 0.8 % (Fig. 3).

**Fig. 4:**
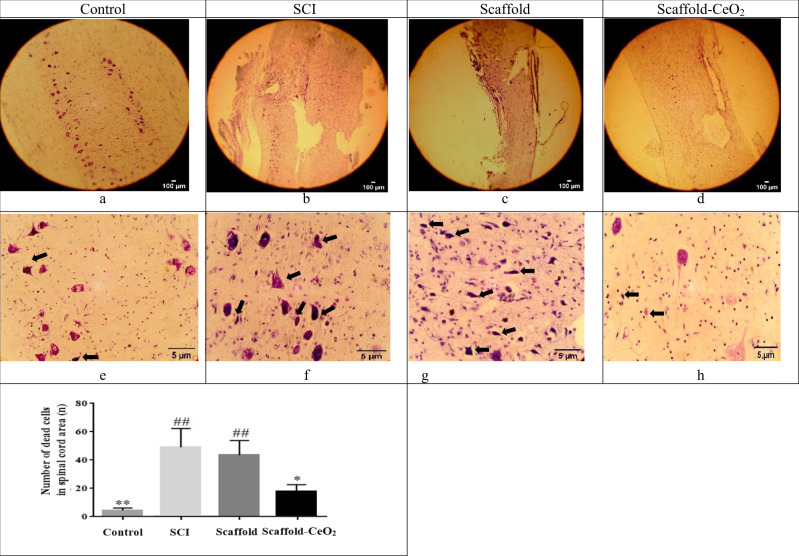
Nissl staining. **a** The normal structure of the spinal cord in the control group and (**b**) Significant structural changes in SCI group. An improvement is observed in structure of spinal cord in (**c**) Scaffold group and more better structure is observed in (**d**) Scaffold-CeO_2_ Group. Nissl bodies is observed in (**e**) very low number in Control group and (**f**) the loss of nerve cells and the emergence of Nissl bodies are visible in the SCI group, **g** In the Scaffold, the number of Nissl bodies was not significantly different from the SCI group, but in the (**h**) Scaffold-CeO_2_ group, a significant difference was observed with the SCI group, while no difference was observed with the control group. *p < 0.05, **p < 0.01, vs. SCI group. ^##^*p* < 0.01, ^###^*p* < 0.001 vs. Control group

The results of the Nissl staining in Fig. 4 shows a small number of Nissl bodies in the control group (4.3 ± 1.6) and a large number of Nissl bodies as well as degenerated neurons with scattered cell arrangement can be seen in the SCI group (49.6 ± 12.4). The difference between the number of dead neurons in the Scaffold group (44.3 ± 9.4) and the SCI group (49.6 ± 12.4) was not significant, but in the Scaffold-CeO_2_ (19.3 ± 3.5) group the number of dead cells was significantly lower than the SCI group.

### The effect of Scaffold-CeO_2_ implant on IBA-1 expression

**Fig. 5:**
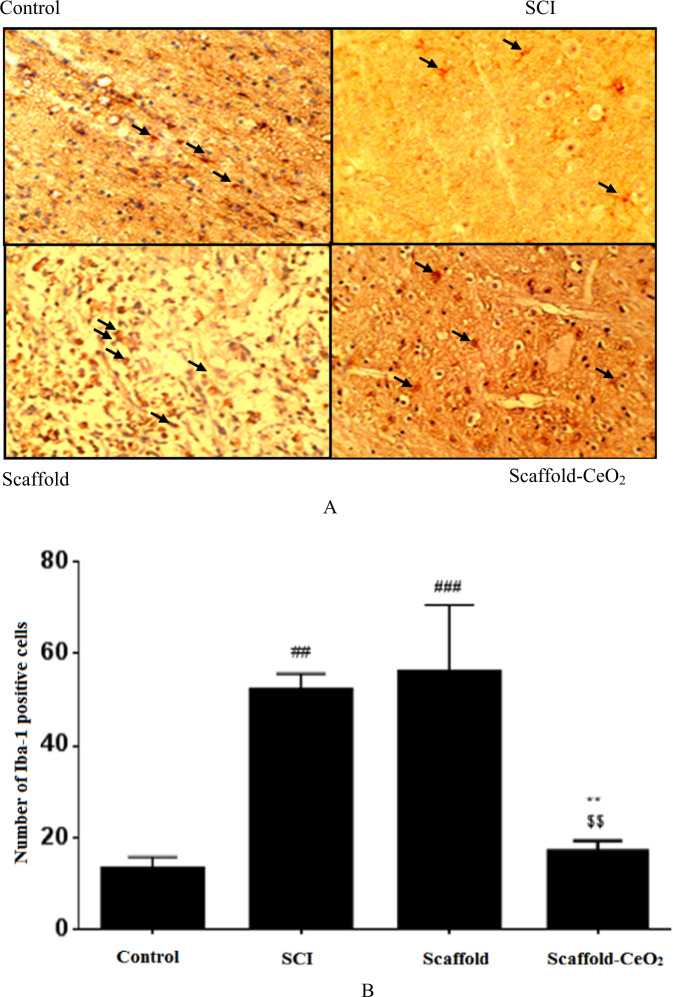
Effect of SCI and implantation of Scaffold and Scaffold-CeO_2_ on expression of Iba-1 in (**A**) Control, Spinal Cord Injury (SCI) group, Scaffold (Sc) group and Scaffold-CeO_2_ group. **B** Quantified data. Data is presented as mean ± SEM (*n* = 3). In each group *p < 0.01 **, vs. SCI group. ^##^*p* < 0.01, ^###^*p* < 0.001, vs. control group.^$$^*p* < 0.01 comparing Scaffold and Scaffold-CeO_2_

As shown in Fig. 5, the expression level of round Iba-1^+^ cells increased in the SCI (53.1 ± 3.1) and Scaffold (57.2 ± 14) groups, while the expression level in the Scaffold-CeO_2_ group (18.3 ± 2.0) decreased and was similar to the control group (13.8 ± 2.4). Inside the spinal cord of the control animals, Iba-1^+^ cells were present throughout the white and gray matter either individually, or in the form of branched cells. In the SCI group, due to the immune response (immune-reactivity) small accumulations of Iba-1^+^ cells in the white matter were created. These clusters did not show the usual branching appearance, but had a large cytoplasm with globoids.

### The effect of Scaffold-CeO_2_ implants on GCSF expression

**Fig. 6:**
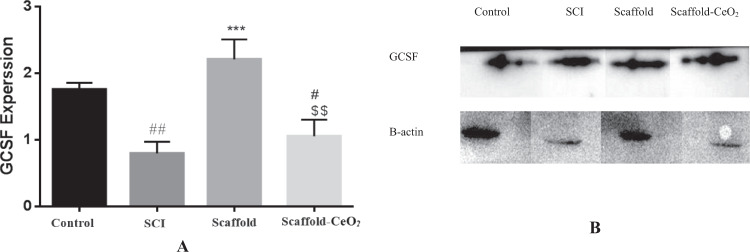
Western blot analysis of GCSF protein expression. This test was performed three times and the intensity of each band was normalized to the corresponding β-actin level. **A** GCSF protein quantification. **B** Protein bands. Data is shown as mean ± SD, *n* = 3. ***, vs. SCI group, ^##^*p* < 0.01, ^#^*p* < 0.05 compared to the control group and ^$$^*p* < 0.01, comparing Scaffold) and Scaffold-CeO_2_

The results in the seventh week showed that GCSF expression was significantly different between the groups. The GCSF expression in SCI group (0.8 ± 0.1) (*p* < 0.001) and Scaffold-CeO_2_ animals (1.1 ± 0.2) were significantly lower compared to the control group (1.8 ± 0.05). The GCSF protein expression levels were significantly increased in Scaffold (2.2 ± 0.1) (*p* < 0.01) compared to the SCI group (0.8 ± 0.1) (*p* < 0.01) (Fig. 6).

### The effect of Scaffold-CeO_2_ implants on Tau expression

**Fig. 7:**
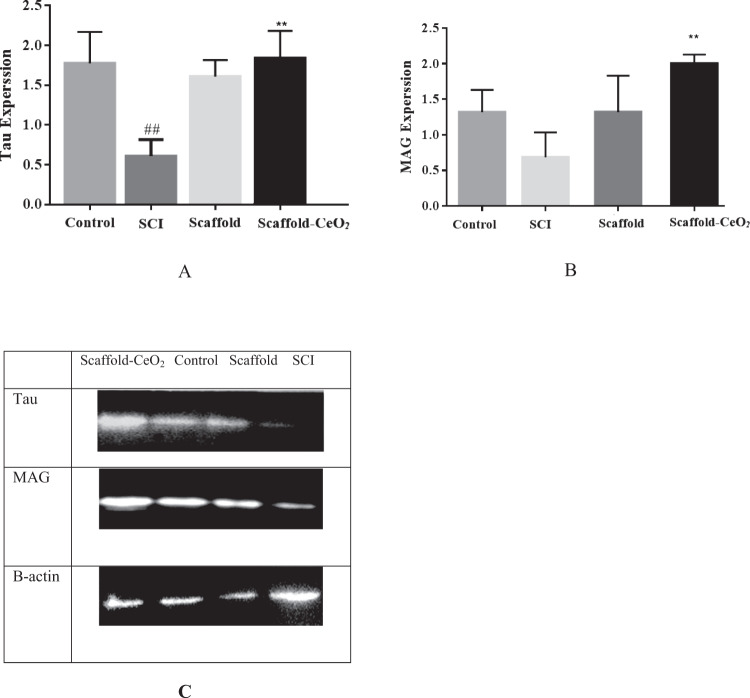
Western blot analysis of Tau and MAG protein expression. This experiment was performed three times and the optical density of each band was normalized to the corresponding β-actin level. **A** Total tau protein. **B** Total MAG protein. **C** Relevant western blot bands. Data is shown as mean ± SD. ^##^*p* < 0.01, ^#^*p* < 0.05 compared with the control group and ^$$$^*p* < 0.001, ^$$^*p* < 0.01, ^$^*p* < 0.05 compared with the group receiving treatment after injury

The results showed a significant difference in Tau expression between the groups at the seventh week. Tau expression was significantly lower in the SCI group (0.8 ± 0.1) compared to the control group (1.77 ± 0.1) (*p* < 0.05). The level of Tau expression in animals in the Scaffold-CeO_2_ group (1.1 ± 0.2) increased significantly compared to the SCI group (0.8 ± 0.1) (*p* < 0.05), and there was no significant difference compared to the control group (1.77 ± 0.1) (Fig. 7A).

### The effect of Scaffold-CeO_2_ implants on MAG expression

SCI induction resulted in a significant difference in total MAG expression compared to the other groups. MAG expression was lower in SCI animals (0.8 ± 0.1) compared to the control group (1.8 ± 0.1), and MAG expression was higher in the Scaffold-CeO_2_ group (1.1 ± 0.2) compared to the SCI group (0.8 ± 0.1) (Fig. 7B).

## Discussion

In this study, SCI on the left side was induced by removing a piece of spinal cord. We investigated the effect of gelatin-PCL containing CeONPs (Scaffold-CeO_2_) on motor recovery and pain relief after SCI. Behavioral changes related to movement and pain in both legs were reported separately. Evaluation of motor changes showed that the right paw was also affected by the left side spinal cord injury and its movement was reduced, which is consistent with previous studies [64, 65]. In the motor function of the left paw, it was observed that the Scaffold alone could not improve movement, but the Scaffold-CeO_2_ significantly improved the left paw movement compared to the SCI and Scaffold groups, although this improvement did not reach the level of the control group. This result is consistent with the results of other studies that showed injection of cerium oxide nanoparticles has helped to improve motor function after spinal cord injury [53, 54].

The result showed at the end of the seventh week, thermal hyperalgesia improved in the left paw, which could indicate the analgesic effect of the Scaffold-CeO_2_ on thermal hyperalgesia. The result of allodynia experiment (mechanical and thermal) also showed the right and left paw of treated animals improved in the seventh week, which indicated the positive effect of Scaffold and Scaffold-CeO_2_ in reducing pain. Our study for the first time showed the effect of CeO_2_NPs in pain relief after spinal cord injury in rat model.

Overall, based on the behavioral experiments, Scaffold-CeO_2_ helped to improve movement in both the injured and the healthy paw, but the scaffold alone did not have this effect on the injured paw. In tolerating heat-induced pain, Scaffold-CeO_2_ treatment also helped significantly in relieving pain, but the Scaffold alone was not able to do this. In mechanical and cold allodynia, the effect of Scaffold-CeO_2_ and Scaffold treatment was similar in both paws and showed a significant difference compared to the SCI group, while it was no different from the control group. Scaffold alone had a positive effect on reducing cold and mechanical pain, but had no effect on improving functional recovery and heat pain tolerance, and the addition of CeO_2_ nanoparticles to the scaffold, in addition to improving cold and mechanical pain tolerance, also improved functional recovery and heat pain tolerance.

Microglia play an important role in CNS defense and tissue repair. In activated microglia, the expression of Iba-1 is increased [66]. Macrophages migrate and release a variety of cytokines, and then become phagocytic to provide a useful environment for promoting the regeneration of sensory axons [66]. In this study, in the SCI and Scaffold groups, the expression of Iba-1 increased, indicating phagocyte activity, but in the Scaffold-CeO_2_ group, it returned to normal, indicating tissue repair. In the spinal cord of control animals, Iba-1-positive cells were ubiquitous throughout the white and gray matter as single cells with a ramified appearance. Analysis of the injured spinal cord showed increased immunoreactivity in addition to small clusters of 3–5 Iba-1-positive cells in the white matter. These aggregates lacked the typical branched appearance and had large and globoid cytoplasmic staining.

Spinal microglial activation plays a major role in producing neuropathic pain following SCI. Evidence has shown that an elevated expression of Iba-1 as a microglial marker persists for at least 14 weeks after L5 spinal hemisection model, while mechanical hypersensitivity decreased. These results indicated that microglia play a role beyond the pain hypersensitivity phase [67]. Other studies have also confirmed a direct relationship between Iba-1^+^ glial cells and pain relief [68]. In our study, Iba-1 increased following SCI, while neuropathic pain (hyperalgesia) also increased, and scaffolds containing CeONPs improved both these measures. Therefore it seems that activated microglia after SCI, in addition to inducing an inhibitory barrier and suppressing the progression of sprouting axons, by releasing inflammatory factors can increase the central sensitivity and subsequently cause and maintain neuropathic pain [60, 69]. Therefore, the reduction after Scaffold-CeO_2_ treatment is a good sign that recovery is progressing.

G-CSF is produce by monocytes, fibroblasts and endothelial cells. G-CSF was initially identified as a major regulator of neutrophil and granulocyte production and modulates the proliferation, survival, maturation and functional activation of these cells [70]. G-CSF prevents the secretion of pro-inflammatory factors, increases the expression of neurotrophic factors and the macrophage phenotype of type 2 [71]. The increase in G-CSF in the Scaffold group can show the increase in the number of fibroblasts in this group. The expression of GCSF decreased in the scaffold-CeO_2_ group, which is not possible to justify with the current knowledge of the researchers of this experiment and requires more observations for conclusion and interpretation.

One of the most important microtubule-associated proteins that contributes to a number of cellular processes, including axonal trafficking, myelination, and synaptic plasticity, and which is also involved in pain perception is Tau protein [72–74]. Following axonal damage, Tau protein is primarily phosphorylated on various amino acids and broken down into smaller fragments. These products can leak into the cerebrospinal fluid or the bloodstream after CNS trauma and act as a biomarker of CNS damage. According to reports, Following SCI, in the first hours and days after SCI, the total amount of Tau in the tissue decreased and the amount of Tau secreted in serum or CSF increased indicating that the process of neuronal death and axonal injury continues [72, 75, 76]. In our study, the amount of Tau in the spinal tissue also decreased in the SCI group, but in the treatment group, the amount of Tau was not significantly different from the control group, which indicates the return of axon stability after receiving treatment after SCI. In healthy neural tissue, tau stabilizes microtubules in cells and is specially abundant in neurons [77]. In agreement with this, we observed high levels of Tau protein in the control group. The amount of Tau in the spinal tissue decreased in the SCI group, but in the Scaffold-CeO_2_ group the amount of Tau was not significantly different from the control group, which indicates the restoration of axonal stability. MAG is a membrane glycoprotein expressed in the oligodendrocyte axon membrane between axons and the inner myelin sheath, and acts to maintain myelinated axons in the adult nervous system. It is interesting to note that MAG plays an important role in axonal growth which depends on the growth stage of the neurons studied. MAG stimulates the growth of immature neurons while preventing the growth of older neurons [78, 79]. +a pioneering study, Filbin et al. explicitly demonstrated the inhibitory role of MAG, as well as the growth promoting effect on newly formed neurons, suggesting that MAG is required for the germination of cortical axons [78]. On the other hand, the role of MAG in axon stabilization and/or axon protection [79, 80]. Therefore, decreased MAG expression in SCI can indicate the damage and separation of myelinated axons in the SCI model, and increased expression in the treatment group in the present study is consistent with the growth of new nerve cells in treated animals.

## Conclusion

The use of CeO_2_ nanoparticles coated on a gelatin- poly (ε-caprolactone) polymer scaffold after SCI, improved motor function, and provided pain relief in animals receiving Scaffold-CeO_2_. Decreased expression of Iba-1 and GCSF and higher expression of Tau and Mag in the SCI Scaffold-CeO_2_ group compared to the SCI group could explain the nerve regeneration as well as pain relief symptoms.

## Supplementary information

Supplementary Information

## Data Availability

Data are available from corresponding authors (FR and AJ) by reasonable request.
