# Author Correction: Study of nerve cell regeneration on nanofibers containing cerium oxide nanoparticles in a spinal cord injury model in rats

**DOI:** 10.1007/s10856-023-06722-6

**Published:** 2023-05-16

**Authors:** Behnaz Rahimi, Zahra Behroozi, Ali Motamednezhad, Maral Jafarpour, Michael R. Hamblin, Ali Moshiri, Atousa Janzadeh, Fatemeh Ramezani

**Affiliations:** 1 Department of basic sciences, Saveh University of Medical Sciences, Saveh, Iran; 2 Physiology Research Center, Institute of Neuropharmacology, Kerman University of Medical Science, Kerman, Iran; 3 Radiation Biology Research Center, Iran University of Medical Sciences, Tehran, Iran; 4 International Campus, Iran University of Medical Sciences, Tehran, Iran; 5 Laser Research Centre, Faculty of Health Science, University of Johannesburg, Doornfontein, 2028 South Africa; 6 Dr. Moshiri Veterinary Clinic, Tehran, Iran; 7 Physiology Research Center, Iran University of Medical Sciences, Tehran, Iran

Correction to: Journal of Materials Science: Materials in Medicine

10.1007/s10856-023-06711-9

The original version of this article unfortunately contained mistakes.

1. The second author Zahra Behroozi had two affiliations listed. Her affiliation is only the affiliation listed as follows: Physiology Research Center, Institute of Neuropharmacology, Kerman University of Medical Science, Kerman, Iran.

2. The full name of the third author is Ali Motamednezhad.

3. Atousa Janzadeh’s academic e-mail address is janzadeh.at@iums.ac.ir

The original article has been corrected.

