# Supplementary material for: Study of nerve cell regeneration on nanofibers containing cerium oxide nanoparticles in a spinal cord injury model in rats
Source: J Mater Sci Mater Med. 2023 Feb 21;34(2):9. doi: 10.1007/s10856-023-06711-9 (PMC9944598; doi:10.1007/s10856-023-06711-9)
Supplement: Supplementary file 1 — Supplementary Information [file 10856_2023_6711_MOESM1_ESM.docx]

|   B |   A |
| --- | --- |
|   D |   C |
| Figure 1S. The effect of Scaffold+Nano immediately after SCI induction on right paw A) motor function (BBB), B) thermal hyperalgesia, C) cold allodynia, D) mechanical allodynia. Data is defined as mean ± SEM (n = 8). In each group * p <0.05, p <0.01 **, *** p <0.001, **** p <0.0001 vs. SCI group. p <0.05 #., ## p <0.01, ### p <0.001, #### p <0.0001 vs. control group. $$ p<0.01 comparing Scaffold and Scaffold+Nano.  SCI: Spinal Cord Injury, SC: scaffold, Sc+Nano: Scaffold+ CeONPs | |
